# Role of PU.1 in MHC Class II Expression via CIITA Transcription in Plasmacytoid Dendritic Cells

**DOI:** 10.1371/journal.pone.0154094

**Published:** 2016-04-22

**Authors:** Ryosuke Miura, Kazumi Kasakura, Nobuhiro Nakano, Mutsuko Hara, Keiko Maeda, Ko Okumura, Hideoki Ogawa, Takuya Yashiro, Chiharu Nishiyama

**Affiliations:** 1 Laboratory of Molecular Biology and Immunology, Department of Biological Science and Technology, Faculty of Industrial Science and Technology, Tokyo University of Science, 6-3-1 Niijuku, Katsushika-ku, Tokyo, 125–8585, Japan; 2 Atopy (Allergy) Research Center, Juntendo University School of Medicine, 2-1-1 Hongo, Bunkyo-ku, Tokyo, 113–8421, Japan; Johns Hopkins University, UNITED STATES

## Abstract

The cofactor CIITA is a master regulator of MHC class II expression and several transcription factors regulating the cell type-specific expression of CIITA have been identified. Although the MHC class II expression in plasmacytoid dendritic cells (pDCs) is also mediated by CIITA, the transcription factors involved in the CIITA expression in pDCs are largely unknown. In the present study, we analyzed the role of a hematopoietic lineage-specific transcription factor, PU.1, in CIITA transcription in pDCs. The introduction of PU.1 siRNA into mouse pDCs and a human pDC cell line, CAL-1, reduced the mRNA levels of MHC class II and CIITA. When the binding of PU.1 to the 3rd promoter of CIITA (pIII) in CAL-1 and mouse pDCs was analyzed by a chromatin immunoprecipitation assay, a significant amount of PU.1 binding to the pIII was detected, which was definitely decreased in PU.1 siRNA-transfected cells. Reporter assays showed that PU.1 knockdown reduced the pIII promoter activity and that three Ets-motifs in the human pIII promoter were candidates of *cis*-enhancing elements. By electrophoretic mobility shift assays, it was confirmed that two Ets-motifs, GGAA (-181/-178) and AGAA (-114/-111), among three candidates, were directly bound with PU.1. When mouse pDCs and CAL-1 cells were stimulated by GM-CSF, mRNA levels of PU.1, pIII-driven CIITA, total CIITA, MHC class II, and the amount of PU.1 binding to pIII were significantly increased. The GM-CSF-mediated up-regulation of these mRNAs was canceled in PU.1 siRNA-introduced cells. Taking these results together, we conclude that PU.1 transactivates the pIII through direct binding to Ets-motifs in the promoter in pDCs.

## Introduction

MHC class II plays essential roles in the adaptive immune system by presenting antigen peptide toward T cell receptor on CD4^+^ T cells. The expression of MHC class II is mainly restricted to thymic epithelial cells and professional antigen-presenting cells, such as dendritic cells (DCs), macrophages, and B cells. In the case of other non-hematopoietic cells, including fibroblasts, epithelial cells, endothelial cells, keratinocytes, and astrocytes, MHC class II expression is induced upon IFN-γ stimulation. The restricted MHC class II expression on professional antigen-presenting cells, thymic epithelial cells, and IFN-γ-stimulated cells reflects the expression profile of a cofactor, class II transactivator (CIITA), which functions as a master regulator for MHC class II [1, 2]. Human *CIITA* and murine *C2ta* genes possesses four (pI, pII, pIII, and pIV) and three (pI, pIIII, and pIV) independent promoters, respectively [1, 3].

The transcription factor PU.1 belongs to the Ets-family, which possesses highly conserved DNA-binding domains termed Ets-domains. PU.1 is expressed in a hematopoietic lineage-specific manner and is involved in the gene expression and development of lymphoid and myeloid cells. PU.1 knockout mice exhibit incomplete hematopoietic cell development, including the abolition of macrophage and B cell production, the delay of neutrophil and T cell development, and the reduction of NK cell and DC production [4–9]. Previous studies including ours showed that PU.1 positively regulates the expression of MHC class II via the transcription of CIITA in conventional DCs (cDCs), B cells, mast cells, and activated T cells [10–16]. Briefly, PU.1 transactivates the pI in cDCs through direct binding to *cis*-enhancing elements in the pI [10, 15], whereas PU.1 transactivates the pIII in Notch1-stimulated mast cells [13], activated human T cells [12], and B cells via proximal region [11] and distal elements including HSS1 [14, 16]. Although it is known that plasmacytoid DCs (pDCs) express CIITA driven from pIII [17], the regulatory mechanism of the pIII in pDCs is largely unknown. On the basis of these observations and considering the presence of PU.1 in pDCs, we hypothesized that PU.1 regulates the pIII in pDCs and is involved in the MHC class II expression in pDCs. In the present study, we demonstrate the important role of PU.1 in the transactivation of CIITA pIII in pDCs.

## Materials and Methods

### Cells and Mice

A human pDC-like cell line, CAL-1 [18], was kindly provided by Dr. Takahiro Maeda (Nagasaki University Graduate School of Biomedical Science) and was maintained in RPMI1640 (Corning, NY) supplemented with 10% heat-inactivated FCS (Biosera, UK), 100 U/ml penicillin, and 100 μg/ml streptomycin. Ten ng/ml recombinant human (rh) GM-CSF (Wako, Osaka, Japan) was added to stimulate the CAL-1 cells. Murine splenic DCs were prepared from BALB/c mice (Japan SLC, Hamamatsu, Japan) as described previously [19] and pDCs were isolated by using the MACS separation system with anti-mouse PDCA-1 MicroBeads (#130-091-965) and an autoMACS pro (Miltenyi Biotech, Tubingen, Germany). Bone marrow-derived pDCs were generated from the femoral and tibial bone marrow cells of BALB/c mice. Briefly, cells were incubated in RPMI 1640 (Sigma-Aldrich, St. Louis, MO) supplemented with 10% heat-inactivated fetal calf serum, 100 U/mL of penicillin, 100 μg/mL streptomycin, 100 μM 2-mercaptoethanol, 10 μM Minimum Essential Medium nonessential amino acid solution, and 100 ng/mL of murine Flt-3L (PeproTech, London, United Kingdom) at 37°C in a humidified atmosphere in the presence of 5% CO_2_ for 6 days. At day 6, PDCA-1^+^ cells were isolated by using a Cell Sorter SH800 (Sony Co., Tokyo, Japan). Mice were sacrificed by cervical dislocation under anesthesia with Isoflurane and Pentobarbital in all mouse experiments. Animal experiments were performed according to the approved guidelines of the Institutional Review Board of Juntendo University School of Medicine, Tokyo, Japan, and of Tokyo University of Science, Tokyo, Japan, and the Animal Care and Use Committees of Tokyo University of Science and of Juntendo University School of Medicine specifically approved this study.

### Knockdown of PU.1 expression by siRNA

**Fig 1:**
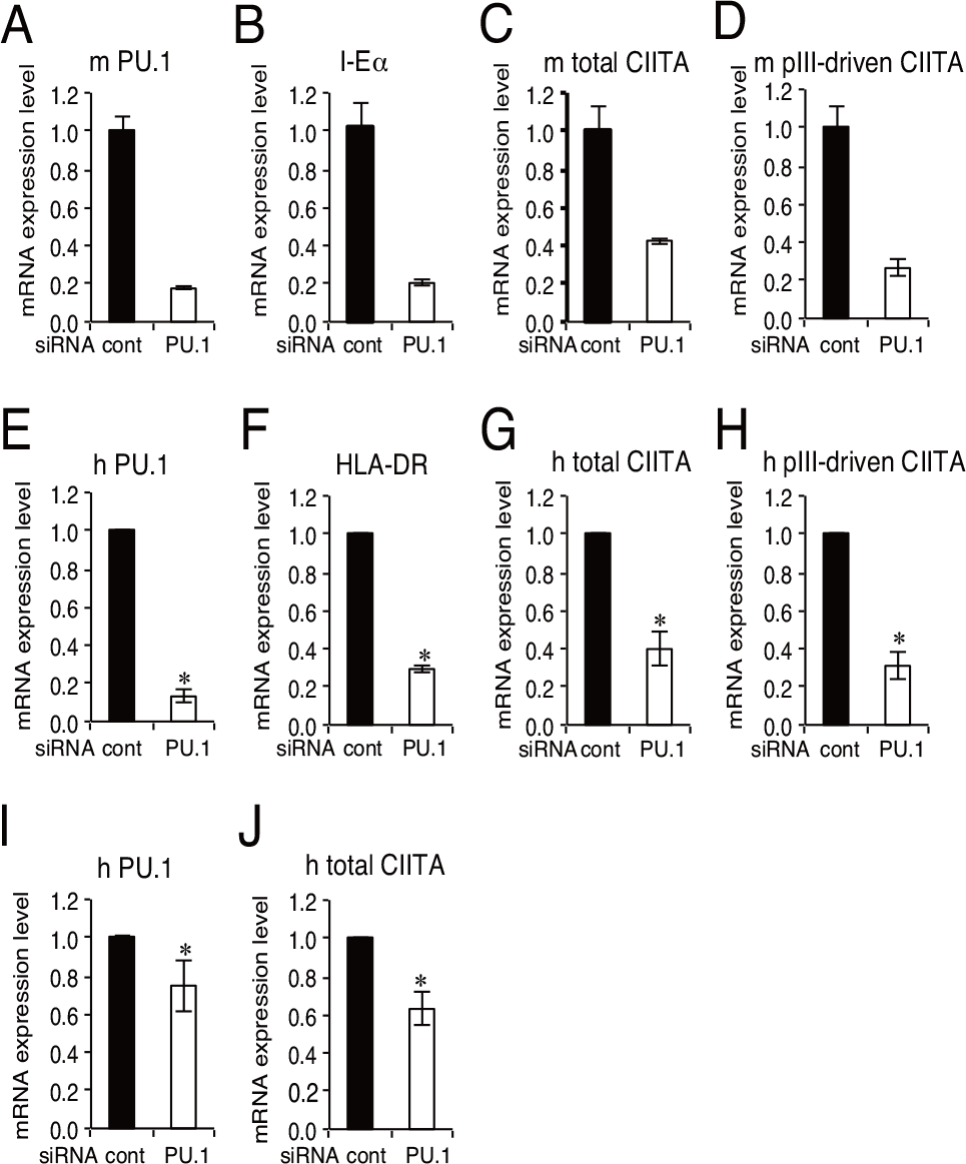
Effect of PU.1 knockdown by siRNA on the mRNA levels of CIITA and MHC class II in mouse pDCs and human pDC cell line. The mRNA expression levels of PU.1 (A, E), MHC class II (B, F), CIITA (C, G), and CIITA specifically driven from pIII (D, H) in mouse PU.1 siRNA-introduced BMpDCs (A-D), or human PU.1 siRNA (Spi1-HSS186060)-introduced CAL-1 cells (E-H) are displayed as the ratio of mRNA levels versus those seen in control siRNA-introduced cells. The mRNA levels of PU.1 (I) and CIITA (J) in CAL-1 cells transfected with another PU.1 siRNA (Spi1-HSS144058) are also displayed. Cells were harvested at 24h (A-D) or 96h (E-J) after siRNA transfection. Open bar, PU.1 siRNA; closed bar, control siRNA. In E-J, data represent means ± SEMs of three independent experiments, and each experiment was performed with triplicate samples. In A-E, the results are expressed as means ± SEMs of triplicate samples, and similar result was obtained in another experiment. *, *p* < 0.05.

PU.1 siRNA (Stealth Select RNAi, Spi1-MSS247676 for mouse PU.1, Spi1-HSS186060 for human PU.1 and Spi1-HSS144058 for human PU.1 in Fig 1I and 1J) and control siRNA (Stealth Negative Control, Med GC no. 45–2001 for Spi1-MSS247676 and Spi1-HSS186060, and Hi GC no. 45–2000 for Spi1-HSS144058) were purchased from Invitrogen (Carlsbad, CA). A total of 5 μl of 20 μM siRNA was introduced into 1 × 10^6^ CAL-1 cells with Neon transfection system (Invitrogen) set at #4 or introduced into 1 × 10^7^ mouse pDCs with a Mouse Dendritic Cell Nucleofector kit (Lonza, Basel, Switzerland) using Nucleofector II (Lonza) set at #X-001.

### Quantification of mRNA by real-time PCR

Total RNA was prepared from cells with an RNeasy kit (QIAGEN, Hilden, Germany) or a Relia Prep RNA Cell Miniprep System (Promega, Madison, WI) and was reverse-transcribed using a Rever Tra Ace qPCR RT kit (TOYOBO, Osaka, Japan) to synthesize cDNA. The mRNA levels of PU.1, HLA-DRα and mouse MHC class II, CIITA, and glyceraldehyde-3-phosphate dehydrogenase (GAPDH) were quantified by using a Step-One Real-Time PCR system (Applied Biosystems) with TaqMan Gene Expression Assays (Applied Biosystems: no. Hs02786711_m1 for human PU.1, Mm01270606_m1 for mouse PU.1, Hs00219575_m1 for HLA-DRα, Mm00772352_m1 for I-Eα, Hs00172106_m1 for human CIITA, Mm00482919_m1 for mouse CIITA, Mm01342720_m1 for mouse CIITA mRNA driven from promoter III (pIII-CIITA), human GAPDH no. 4326317E, and mouse GAPDH no. 4352339E) and TaqMan Universal Master Mix (Applied Biosystems). For the measurement of human pIII-CIITA, the following primers and probe were originally constructed using the customized service of Applied Biosystems: forward primer, 5’-GCTGGGATTCCTACACAATGC-3’; reverse primer, 5’-TCTCCAGCCAGGTCCATCTG-3’; and probe, 5’-FAM-CCCAAGGCAGCTCA-MGB-3’. The expression level of each mRNA was evaluated relative to that of GAPDH by calculation of cycle threshold (Ct) values as described previously [20].

### Luciferase reporter assay

A series of reporter plasmids carrying the CIITA-pIII promoter region just upstream of the luciferase gene in pGL4-Basic (Promega) were generated by using PCR and site-directed mutagenesis. The nucleotide sequences of synthesized oligonucleotides that were used as primers are listed in S1 Table. CAL-1 cells (5 × 10^5^) were transfected with 2 μg of pGL4.10-based reporter plasmid, and 2 ng of pRL-CMV (Promega) using Neon transfection system set at #4. Determination of luciferase activity was performed as described previously by using a luminomator, Micro Lumat Plus (Berthold Technologies, Bad Wildbad, Germany) or 1420 Luminescence Counter ARVO Light (Perkin Elmer) [20]. *Renilla* luciferase activity driven by pRL-CMV was used as an internal control to normalize the transfection efficiency.

### Chromatin immunoprecipitation (ChIP) assay

ChIP assays were performed as described previously using anti-PU.1 goat IgG (D-19, no. sc-5040, Santa Cruz Biotechnology, Santa Cruz, CA) and goat IgG (no. 02–6202, Invitrogen) [20, 21]. The amount of chromosomal DNA including the CIITA-pIII promoter was determined by quantitative real-time PCR using the primers listed in S2 Table, and the ratio of immunoprecipitated DNA was calculated as described previously [20, 21].

### Electrophoretic mobility shift assay (EMSA)

Double-stranded probes were prepared by annealing synthesized oligonucleotides and their complementary oligonucleotides, which were FITC-labeled at the 5’-end. Preparation and electrophoresis of the probe/protein mixture were performed as described previously [10, 22]. Briefly, PU.1 protein was prepared with a TNT T7 Quick coupled transcription/translation system (Promega). The reaction mixtures were subjected to electrophoresis with a native 4% polyacrylamide gel at 200V for 1.5 ~ 2.0 h in 0.5× TBE buffer. Fluorescence was detected by using an image analyzer, Typhoon FLA 7000 (GE Healthcare).

### Statistical analysis

Statistical analysis was performed using a two-tailed Student’s t-test with *p* values <0.05 considered significant.

## Results

### Effect of PU.1 siRNA on the mRNA levels of CIITA and MHC class II in pDCs

To evaluate the effect of PU.1 suppression on MHC class II expression in pDCs, PU.1 siRNA was introduced into mouse bone marrow-derived pDCs (BMpDCs). When siRNA was introduced into cells by using our optimized method, PU.1 mRNA level in PU.1 siRNA-introduced pDCs was reduced to approximately 10% of that of control siRNA-introduced cells (Fig 1A). Under these experimental conditions, mRNA levels of MHC class II and CIITA were significantly decreased compared with those of control cells (Fig 1B and 1C). In addition, we determined the amount of CIITA mRNA driven from pIII (hereafter referred to as pIII-CIITA) by using a specific primer set for the transcript from pIII and found that PU.1 knockdown suppressed pIII-CIITA mRNA level (Fig 1D). We also used a human pDC cell line, CAL-1, for PU.1 knockdown experiment, and found that HLA-DRα (F), CIITA (G), and pIII-CIITA (H) was significantly reduced in PU.1 knockdown condition (E) as observed in mouse primary pDCs. When another PU.1 siRNA of different sequence was used to exclude the possibility of off-target effect, significant reduction of CIITA mRNA level (J) in parallel with PU.1 knockdown level (I) was observed. These results suggest that PU.1 is involved in the transcription of MHC class II and CIITA, including pIII-CIITA in both mouse pDCs and human pDC cell line.

### PU.1 binds to the CIITA-pIII promoter on chromosomal DNA in pDCs

**Fig 2:**
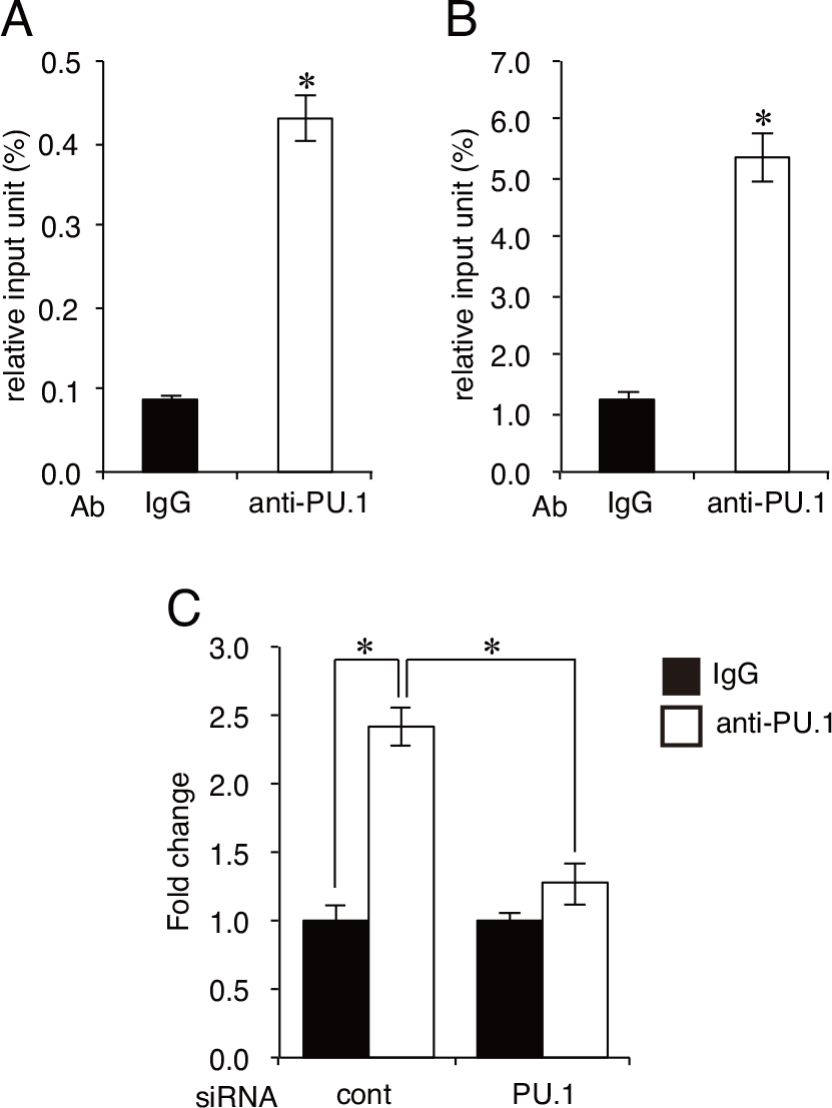
Quantitative analysis of PU.1 binding to the pIII in CAL-1 and murine splenic pDCs. **A and B.** The amount of chromosomal DNA immunprecipitated with anti-PU.1 Ab (open bar, anti-PU.1) or control IgG Ab (closed bar, IgG) in CAL-1 cells (A) and murine splenic pDCs (B). Binding degree is expressed as a percentage of the input for each ChIP assay. **C.** The amount of PU.1 binding to the pIII in PU.1 siRNA-introduced CAL-1 cells (PU.1) or control siRNA-introduced cells (cont.). Cells were harvested at 48h after siRNA transfection. Binding level of PU.1 (open bar) is expressed as fold change against that of control IgG (closed bar). Data represent means ± SEMs of three independent experiments. Each experiment was performed with duplicate samples. *, *p* < 0.05.

Several studies including ours reported that PU.1 is involved in MHC class II expression through the transactivation of CIITA; briefly, PU.1 transactivates the pI promoter in cDC [10, 15], and the pIII promoter in B cells [11, 14, 16] and in mast cells [13]. In contrast, the involvement of PU.1 in pIII function in pDCs is largely unknown. Therefore, a ChIP assay was performed to examine whether PU.1 is recruited to the pIII region on the chromosomal DNA in pDCs. The amount of DNA immunoprecipitated with the anti-PU.1 Ab was significantly higher than that of control Ab in the experiment using CAL-1 cells (Fig 2A). Similarly, when mouse pDCs prepared from spleen were analyzed, a significant amount of the pIII promoter region was immunoprecipitated with anti-PU.1 Ab (Fig 2B). These results indicate that PU.1 binds to the pIII promoter region on chromosomal DNA, which is a common observation between human and mouse, and between the cell line and primary cells.

When the PU.1 expression level was suppressed by PU.1 siRNA, CIITA mRNA driven from the pIII promoter was markedly reduced, as shown in Fig 1. Therefore, we examined whether the level of PU.1 binding to the pIII promoter region was decreased in PU.1 siRNA-introduced cells. As shown in Fig 2C, the amount of PU.1 binding to the pIII promoter region was definitely reduced in PU.1 siRNA-introduced cells, whereas a significant amount of PU.1 binding to the pIII promoter region was detected in control siRNA-introduced cells. These results suggest that the decrease of PU.1 binding to the pIII promoter is associated with the reduced mRNA levels of CIITA and MHC class II in PU.1 siRNA-introduced cells.

### Identification of the *cis*-enhancing elements in the human CIITA-pIII promoter

**Fig 3:**
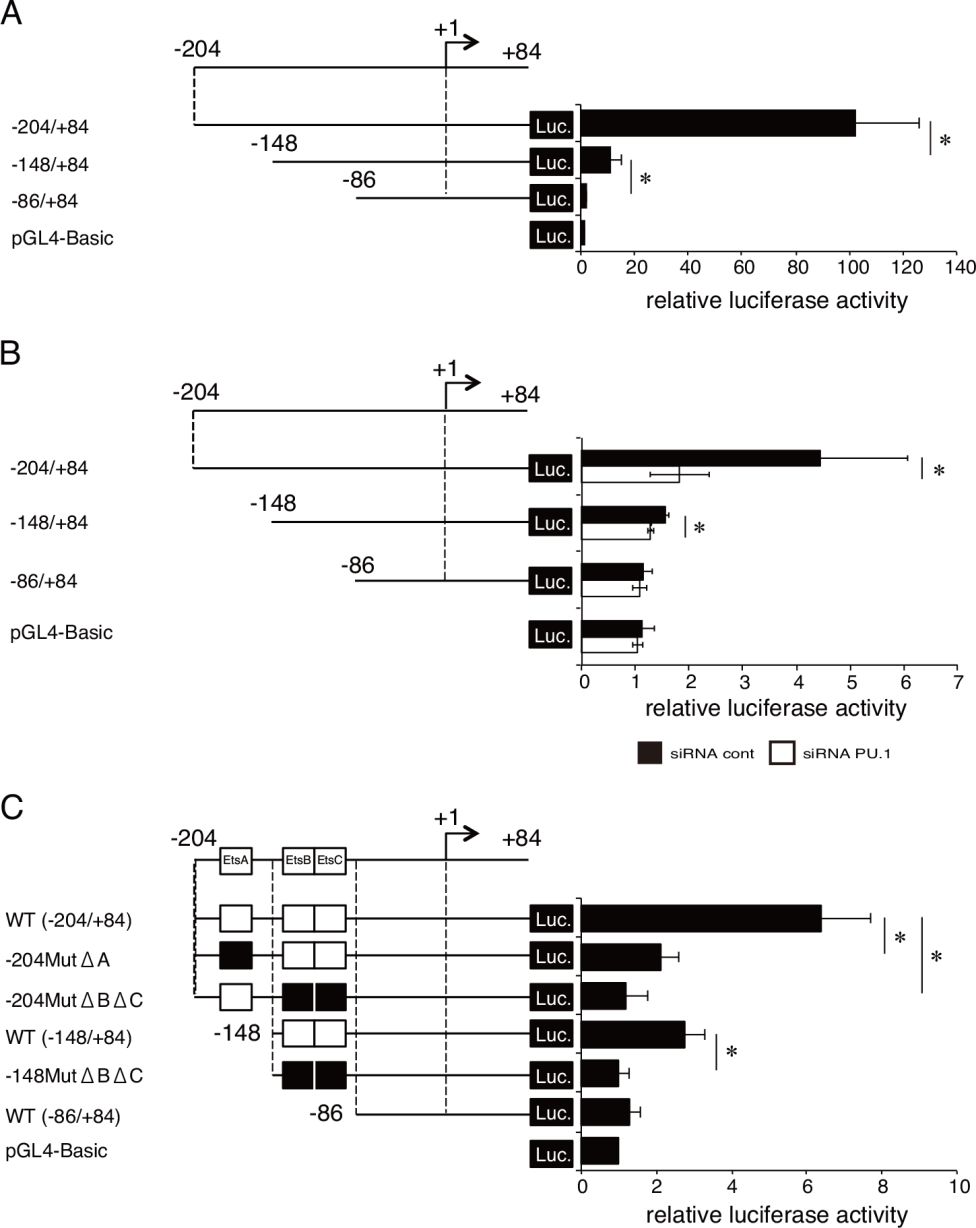
Luciferase activity driven by the human pIII promoter in CAL-1 cells. **A-C.** Luciferase assays performed with various lengths of the human pIII promoters (A), the pIII promoters in the presence of PU.1 siRNA (open bar) or control siRNA (closed bar) (B), and the mutant promoters lacking Ets-motif(s) (C). Data represent means ± SEMs of three independent experiments. Each experiment was performed with triplicate samples. *, *p* < 0.05.

The ChIP assays were performed with primer sets targeting -230/-101 of the human pIII promoter and -200/-125 of the mouse pIII promoter (Fig 2). Several PU.1-responsive sequences are present in this region, suggesting that PU.1 transactivates the pIII promoter through direct binding to the promoter via these *cis*-enhancing element(s). To evaluate the effect of PU.1 on the pIII promoter function and to identify the *cis*-enhancing element(s) in the pIII promoter, reporter assays were performed (Fig 3A, 3B and 3C). Luciferase activity derived from CAL-1 transfected with a reporter plasmid carrying -148/+84 was significantly lower than that of -204/+84, and deletion of the -148/-87 region further reduced the luciferase activity to the basal level, as observed in promoter-less control plasmid (Fig 3A), suggesting that *cis*-enhancing elements are located in -204/-149 and -148/-87. When PU.1 siRNA was introduced into CAL-1 cells with reporter plasmids, luciferase activity driven by the pIII promoter (-204/+84 or -148/+84) was significantly reduced in compared with that of control siRNA co-transfection (Fig 3B). From this result it is confirmed that PU.1 is involved in the pIII promoter through -148/-87 region as a positive regulator. We found one and two typical Ets-motifs in -204/-149 and in -148/-87, respectively, and then generated additional reporter plasmids, in which nucleotide replacements were introduced for the lack of Ets-motif(s), to evaluate the involvement of Ets-motifs in promoter activity. As shown in Fig 3C, two mutant promoters based on the -204/+84 region lacking Ets-A or Ets-B/Ets-C exhibited significantly reduced transcriptional activity compared with that of the wild-type promoter of -204/+84. Mutation at Ets-B/Ets-C in the -148/+84 promoter downregulated the transcriptional activity to a level as low as those of the -86/+84 region and of the promoter-less control (Fig 3C). These results demonstrate that PU.1 is involved in the pIII promoter as positive regulator and that Ets-motifs at -181/-178, -114/-111, and -108/-105 are candidate *cis*-enhancing elements essential for transcriptional activity of the pIII promoter.

### PU.1 directly binds to the identified *cis*-enhancing elements on the human CIITA-pIII promoter

**Fig 4:**
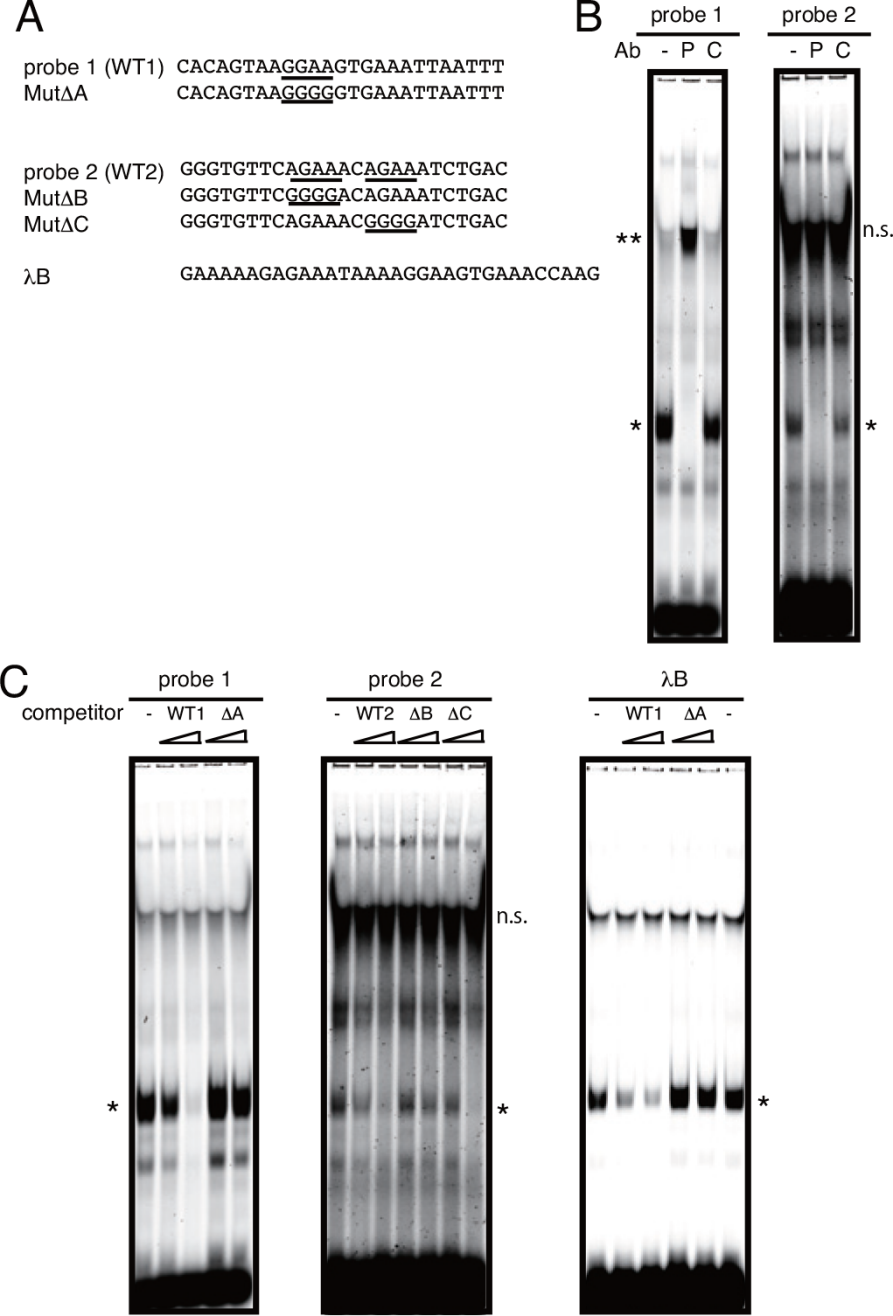
Direct binding of PU.1 to Ets-motifs in the human pIII promoter. **A.** Nucleotide sequences of probes and competitive oligonucleotides used for EMSAs. **B and C.** EMSA profiles. Anti-PU.1 Ab (P) or control Ab (C) was used to identify the specific band composed of PU.1 and probe DNA (B). EMSA with an excess amount of competitors (a 1- or 10-fold molar concentration of probe 1 or 2) was performed to identify PU.1-binding sites (C). λB, which is previously published element bound with PU.1 [23], was mixed with a 15- or 30-fold concentration of competitors. Specific bands corresponding to complexes of PU.1 and the probe are marked with an asterisk. Super-shift band corresponding to complexes of PU.1, the probe, and anti-PU.1 Ab are marked with double asterisks. n.s., non-specific bands detected in protein mixture without probe DNA.

The above-mentioned results indicated that PU.1 binds to the pIII promoter region, in which three PU.1-responsive *cis*-elements are located. Therefore, an EMSA was performed to determine whether PU.1 directly binds to the pIII promoter. When the *in vitro*-translated PU.1 protein was mixed with the FITC-labeled double-stranded -189/-165 region (Fig 4A, probe 1), a specific band appeared on an electrophoretic gel, which disappeared in the presence of anti-PU.1 Ab (Fig 4B left, asterisk), with the appearance of a new lower-mobility band (super-shift band, double asterisk), but it did not disappear in the presence of the control Ab. In the case of the -122/-98 region (Fig 4A, probe 2), a similar specific band that appeared in the lane containing the PU.1/DNA-complex but disappeared in the lane containing the PU.1/DNA/anti-PU.1 antibody-complex (Fig 4B right, asterisk) was observed, although a super-shift band was not detected, probably due to the low density of the band. Next, EMSAs were performed with competitive oligonucleotides for further identification of the PU.1-binding sequences in the -189/-165 region and in the -122/-98 region. The specific band shift corresponding to the PU.1/probe 1-complex disappeared upon the addition of wild-type competitor (WT1), whereas this specific band remained in the presence of the mutant competitor Mut∆A (Fig 4C, left), suggesting that PU.1 binds to GGAA (-181/-178). In the case of the competition assay for probe 2, the specific band disappeared upon the addition of wild-type competitor (WT2) or mutant competitor Mut∆C, but did not disappear in the presence of mutant competitor Mut∆B (Fig 4C, center). The probe λB, which was previously published to form complex with PU.1, was used as positive control [23], and the intensity of the specific band corresponding to PU.1/λB was decreased in the presence of competitor WT1 but not ∆A (Fig 4C, right). From these results, it is concluded that PU.1 directly binds to the pIII promoter via Ets-A (GGAA, -181/-178) and Ets-B (AGAA, -114/-111).

### GM-CSF stimulation up-regulates the expression of CIITA and MHC class II through the increase of the expression and *cis*-element-binding of PU.1

**Fig 5:**
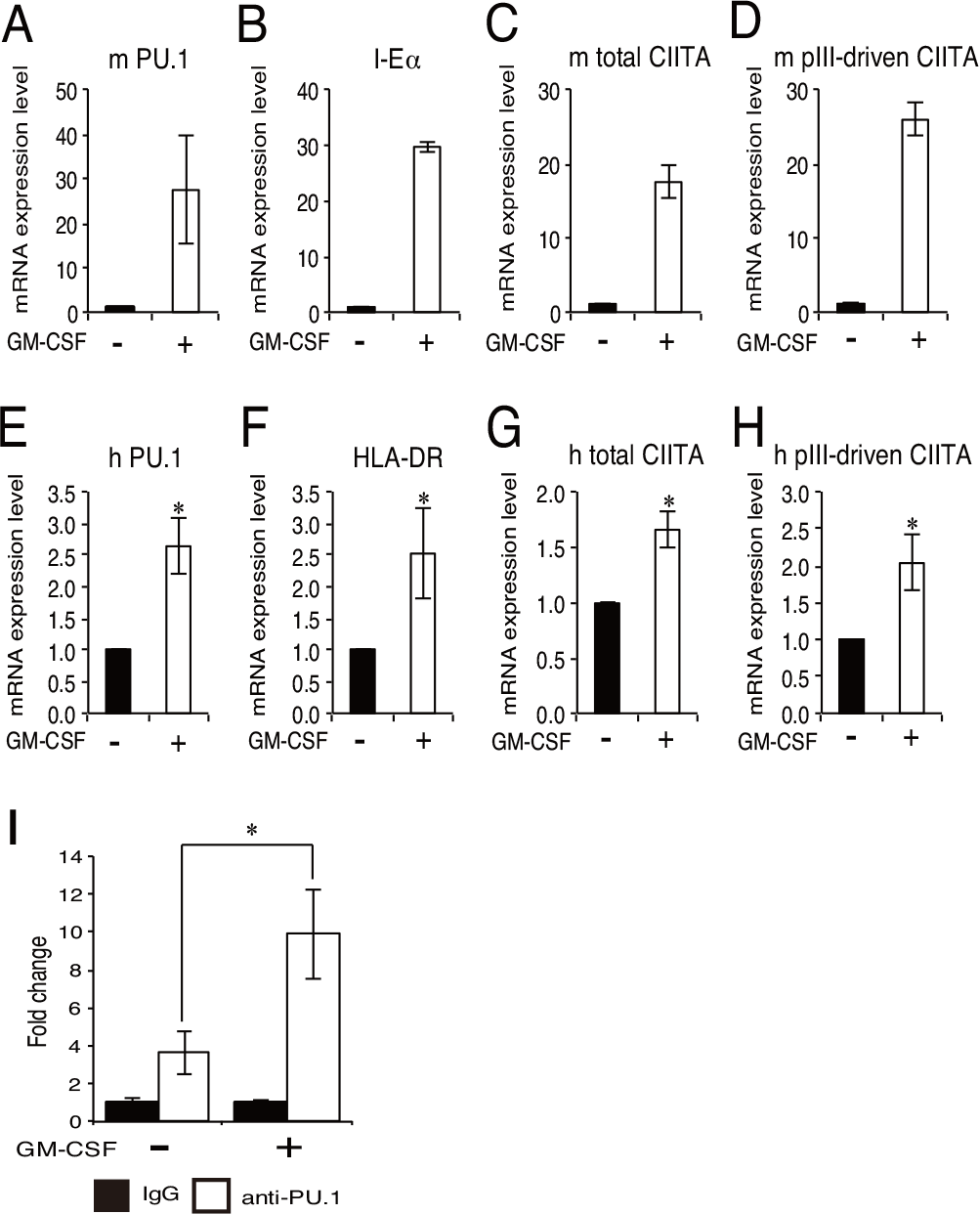
Effect of GM-CSF stimulation on the expression of PU.1, CIITA, and MHC class II, and on the recruitment of PU.1 to the pIII. **A-H.** Quantitative real-time PCR analysis of the mRNA expression of PU.1 (A, E), MHC class II (B, F), total CIITA (C, G), and CIITA driven by pIII (D, H) in mouse splenic pDCs (A-D) or CAL-1 cells (E-H) with (+) or without (-) GM-CSF stimulation. Mouse pDCs and CAL-1 cells were stimulated with 20 ng/ml mGM-CSF for 24h and 10 ng/ml hGM-CSF for 72h, respectively. **I.** The amount of PU.1 binding to the pIII in GM-CSF-stimulated CAL-1 cells (+) or control cells (-) was determined by a ChIP assay. Binding level of PU.1 (open bar) is expressed as fold change against that of control IgG (closed bar). In E-I, data represent means ± SEMs of three independent experiments performed with duplicate samples. In A-E, the results are expressed as means ± SEMs of triplicate samples, and similar result was obtained in another experiment. *, *p* < 0.05.

**Fig 6:**
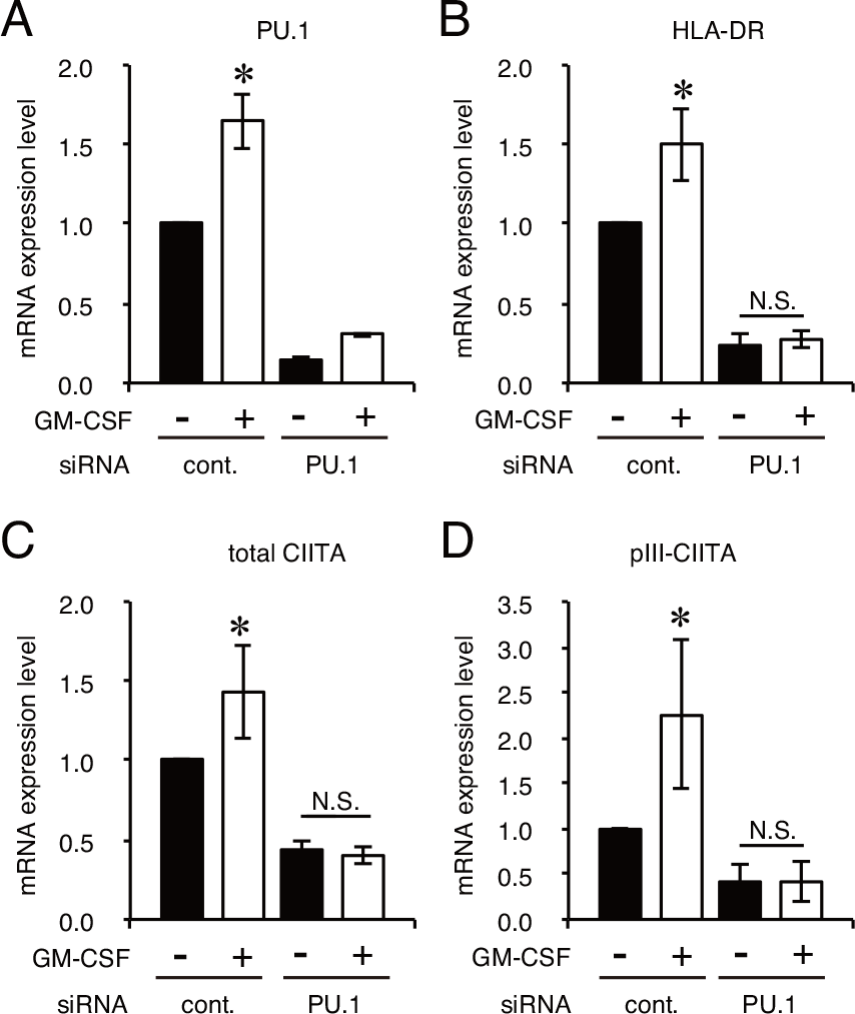
PU.1 knockdown cancels GM-CSF-mediated increase of mRNAs. **A-D.** Quantitative real-time PCR analysis of the mRNA expression of PU.1 (A), HLA-DRα (B), total CIITA (C), and CIITA driven by pIII (D) in PU.1 siRNA (PU.1)- or control siRNA (cont.)-introduced CAL-1 cells. Cells were incubated for 24h after siRNA transfection and cultured for additional 72h with GM-CSF stimulation. The results are expressed as means ± SEMs of three independent experiments. Each experiment was performed with duplicate samples. *, *p* < 0.05.

It has been reported that GM-CSF stimulation up-regulates the expression of CD80 and CD86 in CAL-1 cells [18]. In addition, we previously found that the expression of CD80 and CD86 is critically regulated by PU.1 in DCs [21]. Therefore, we hypothesized that GM-CSF stimulation increases the PU.1 expression and/or function in DCs. When mouse pDCs were incubated in the presence of GM-CSF, as expected, the PU.1 mRNA level was dramatically up-regulated (Fig 5A). In accordance with this increase, the mRNA levels of I-E, total CIITA, and pIII-CIITA were also increased (Fig 5B, 5C and 5D). Similarly, PU.1 mRNA level in GM-CSF-stimulated CAL-1 cells was more than twofold higher than that in non-stimulated cells (Fig 5E) with significant increase of the mRNA levels for HLA-DRα, total CIITA, and pIII-driven CIITA (Fig 5F, 5G and 5H). Under these experimental conditions, the relative PU.1-binding level toward the pIII region was significantly increased in GM-CSF-stimulated cells (Fig 5I). Furthermore, the GM-CSF-mediated increases of mRNA levels for total CIITA, pIII-CIITA, and HLA-DRα were canceled in PU.1 siRNA-introduced CAL-1 cells, whereas control siRNA-introduced cells still exhibited the mRNA response against GM-CSF stimulation (Fig 6). On the basis of these results, it is concluded that enhanced recruitment of PU.1 toward the pIII promoter up-regulates the transcription of CIITA and subsequently MHC class II in GM-CSF-stimulated cells.

## Discussion

CIITA is a master regulator for the expression of MHC class II, whose expression is largely restricted in professional antigen-presenting cells (DCs, macrophages, and B cells), thymic epithelial cells, and IFN-γ-stimulated non-hematopoietic cells. Four (pI, pII, pIII, and pIV) and three (pI, pIII, and pIV) promoters were found in the human *CIITA* and mouse *C2ta* genes, respectively. Initially, pI, pIII, and pIV were known to be mainly used in DCs, B cells, and IFN-γ-stimulated cells, respectively [3, 24, 25]. Several transcription factors have been identified for the regulation of pIII and pIV [1]: STAT1, E47, PU.1, IRF4, AML2/3, CREB, and BLIMP1 for pIII, and STAT1, USF1, IRF1, and IRF2 for pIV. In our previous research focusing on the role of a hematopoietic lineage-specific transcription factor, PU.1, in gene regulation and the development of DCs [10, 21, 26], we found that PU.1 siRNA suppressed MHC class II on DCs [21] and revealed that PU.1 transactivates the first promoter, pI, of CIITA in cDCs, resulting in the subsequent expression of MHC class II [10], whereas PU.1 directly transactivates the promoters of CD80 and CD86 [21], and TNF-α [26]. In contrast to the pI in cDCs, although it is well known that pDCs mainly use the pIII for the expression of CIITA [17], the regulatory mechanism of the pIII in pDCs has not been elucidated. Therefore, in the present study, we investigated the role of PU.1 in the function of the pIII in pDCs. First, we confirmed that the expression of MHC class II, CIITA, and pIII-CIITA was decreased in a PU.1 siRNA-introduced mouse pDCs and human pDC cell line (Fig 1), suggesting that PU.1 is involved in the function of pIII. Following detailed promoter analyses by using ChIP assay (Fig 2), reporter assay (Fig 3), and EMSA (Fig 4) demonstrated that PU.1 binds to the critical *cis*-enhancing elements identified in the pIII. When PU.1 expression was suppressed by siRNA, the binding level of PU.1 to the pIII and the CIITA mRNA level were decreased (Figs 1 and 2). In addition, the binding level of PU.1 to the pIII and the CIITA mRNA level were increased with the up-regulation of PU.1 in GM-CSF-stimulated mouse pDCs and CAL-1 (Fig 5), which was canceled by PU.1 knockdown (Fig 6), indicating the association between PU.1 binding level on the pIII and CIITA transcription level. Taking these results together, we conclude that PU.1 regulates the pIII of CIITA in pDCs through direct binding to the promoter and that the role of PU.1 in pIII function is a common observation between human and mouse, and between the pDC cell line and primary pDCs.

PU.1 possesses the potential to transactivate all of the three CIITA promoters. It also positively regulates pI function as a transcription factor and by affecting histone modification in cDCs [10]. PU.1 transactivates the pIII through direct binding to proximal region [11] in B cells, activated human T cells [12], and Notch-stimulated mast cells [13]. PU.1 is also involved in the pIII function through binding to long-distance regulatory elements on CIITA gene [14, 16]. In addition, we found that enforced expression of PU.1 by using retrovirus vector definitely induces the expression of MHC class II in mast cells via the strong activation of the pIV [27, 28]. It would be interesting to understand the molecular mechanism of distinguished recruitment of PU.1 toward CIITA promoters in a cell type- and/or cell condition-specific manner. IRF4 and IRF8 might be candidates that determine the direction of PU.1 because PU.1 functions not only as a monomeric transcription factor but also as a complex forming dimers with IRF4 or IRF8. In the present experimental conditions, siRNAs for IRF4 and IRF8 did not suppress CIITA expression (data not shown), whereas PU.1 and IRF4 synergistically transactivated the pIII in B cells [11]. Further detailed analyses for the identification of other molecules regulating the CIITA promoters by using various MHC class II-expressing cells will be required to reveal the mechanism that determines the direction of PU.1 and the promoter usage of the CIITA gene.

## Supporting Information

S1 Table Nucleotide sequences of synthesized oligonucleotides for generation of reporter plasmids carrying the human CIITA-pIII promoters by using PCR and site-directed mutagenesis. (DOCX)

S2 Table Nucleotide sequences of synthesized oligonucleotides for ChIP assay. (DOCX)

## Abbreviations

ChIP: Chromatin immunoprecipitation
cDCs: conventional dendritic cells
EMSA: Electrophoretic mobility shift assay
pDCs: plasmacytoid dendritic cells

## References

[1] Reith W, LeibundGut-Landmann S, Waldburger JM. Regulation of MHC class II gene expression by the class II transactivator. Nat Rev Immunol. 2005;5(10):793–806. 10.1038/nri1708 . 16200082

[2] Reith W, Mach B. The bare lymphocyte syndrome and the regulation of MHC expression. Annu Rev Immunol. 2001;19:331–73. 10.1146/annurev.immunol.19.1.331 . 11244040

[3] Muhlethaler-Mottet A, Otten LA, Steimle V, Mach B. Expression of MHC class II molecules in different cellular and functional compartments is controlled by differential usage of multiple promoters of the transactivator CIITA. EMBO J. 1997;16(10):2851–60. 10.1093/emboj/16.10.2851 9184229 PMC1169893

[4] Scott EW, Simon MC, Anastasi J, Singh H. Requirement of transcription factor PU.1 in the development of multiple hematopoietic lineages. Science. 1994;265(5178):1573–7. . 8079170 10.1126/science.8079170

[5] McKercher SR, Torbett BE, Anderson KL, Henkel GW, Vestal DJ, Baribault H, et al Targeted disruption of the PU.1 gene results in multiple hematopoietic abnormalities. EMBO J. 1996;15(20):5647–58. 8896458 PMC452309

[6] Scott EW, Fisher RC, Olson MC, Kehrli EW, Simon MC, Singh H. PU.1 functions in a cell-autonomous manner to control the differentiation of multipotential lymphoid-myeloid progenitors. Immunity. 1997;6(4):437–47. . 9133423 10.1016/s1074-7613(00)80287-3

[7] Anderson KL, Perkin H, Surh CD, Venturini S, Maki RA, Torbett BE. Transcription factor PU.1 is necessary for development of thymic and myeloid progenitor-derived dendritic cells. J Immunol. 2000;164(4):1855–61. . 10657634 10.4049/jimmunol.164.4.1855

[8] Guerriero A, Langmuir PB, Spain LM, Scott EW. PU.1 is required for myeloid-derived but not lymphoid-derived dendritic cells. Blood. 2000;95(3):879–85. . 10648399

[9] Colucci F, Samson SI, DeKoter RP, Lantz O, Singh H, Di Santo JP. Differential requirement for the transcription factor PU.1 in the generation of natural killer cells versus B and T cells. Blood. 2001;97(9):2625–32. . 11313251 10.1182/blood.v97.9.2625

[10] Kitamura N, Yokoyama H, Yashiro T, Nakano N, Nishiyama M, Kanada S, et al Role of PU.1 in MHC class II expression through transcriptional regulation of class II transactivator pI in dendritic cells. J Allergy Clin Immunol. 2012;129(3):814–24 e6. 10.1016/j.jaci.2011.10.019 . 22112519

[11] van der Stoep N, Quinten E, Marcondes Rezende M, van den Elsen PJ. E47, IRF-4, and PU.1 synergize to induce B-cell-specific activation of the class II transactivator promoter III (CIITA-PIII). Blood. 2004;104(9):2849–57. 10.1182/blood-2004-03-0790 . 15242870

[12] Holling TM, van der Stoep N, Quinten E, van den Elsen PJ. Activated human T cells accomplish MHC class II expression through T cell-specific occupation of class II transactivator promoter III. J Immunol. 2002;168(2):763–70. . 11777970 10.4049/jimmunol.168.2.763

[13] Nakano N, Nishiyama C, Yagita H, Koyanagi A, Ogawa H, Okumura K. Notch1-mediated signaling induces MHC class II expression through activation of class II transactivator promoter III in mast cells. J Biol Chem. 2011;286(14):12042–8. 10.1074/jbc.M110.138966 21321116 PMC3069407

[14] Yoon H, Boss JM. PU.1 binds to a distal regulatory element that is necessary for B cell-specific expression of CIITA. J Immunol. 2010;184(9):5018–28. 10.4049/jimmunol.1000079 20363966 PMC3472449

[15] Smith MA, Wright G, Wu J, Tailor P, Ozato K, Chen X, et al Positive regulatory domain I (PRDM1) and IRF8/PU.1 counter-regulate MHC class II transactivator (CIITA) expression during dendritic cell maturation. J Biol Chem. 2011;286(10):7893–904. 10.1074/jbc.M110.165431 21216962 PMC3048676

[16] Lohsen S, Majumder P, Scharer CD, Barwick BG, Austin JW, Zinzow-Kramer WM, et al Common distal elements orchestrate CIITA isoform-specific expression in multiple cell types. Genes Immun. 2014;15(8):543–55. 10.1038/gene.2014.49 25101797 PMC4257854

[17] LeibundGut-Landmann S, Waldburger JM, Reis e Sousa C, Acha-Orbea H, Reith W. MHC class II expression is differentially regulated in plasmacytoid and conventional dendritic cells. Nat Immunol. 2004;5(9):899–908. 10.1038/ni1109 . 15322541

[18] Maeda T, Murata K, Fukushima T, Sugahara K, Tsuruda K, Anami M, et al A novel plasmacytoid dendritic cell line, CAL-1, established from a patient with blastic natural killer cell lymphoma. Int J Hematol. 2005;81(2):148–54. . 15765784 10.1532/ijh97.04116

[19] Yashiro T, Kubo M, Ogawa H, Okumura K, Nishiyama C. PU.1 Suppresses Th2 Cytokine Expression via Silencing of GATA3 Transcription in Dendritic Cells. PLoS One. 2015;10(9):e0137699 10.1371/journal.pone.0137699 26361334 PMC4567381

[20] Maeda K, Nishiyama C, Tokura T, Nakano H, Kanada S, Nishiyama M, et al FOG-1 represses GATA-1-dependent FcepsilonRI beta-chain transcription: transcriptional mechanism of mast-cell-specific gene expression in mice. Blood. 2006;108(1):262–9. 10.1182/blood-2005-07-2878 . 16522818

[21] Kanada S, Nishiyama C, Nakano N, Suzuki R, Maeda K, Hara M, et al Critical role of transcription factor PU.1 in the expression of CD80 and CD86 on dendritic cells. Blood. 2011;117(7):2211–22. 10.1182/blood-2010-06-291898 . 21119111

[22] Baba Y, Maeda K, Yashiro T, Inage E, Kasakura K, Suzuki R, et al GATA2 is a critical transactivator for the human IL1RL1/ST2 promoter in mast cells/basophils: opposing roles for GATA2 and GATA1 in human IL1RL1/ST2 gene expression. J Biol Chem. 2012;287(39):32689–96. 10.1074/jbc.M112.374876 22865859 PMC3463314

[23] Eisenbeis CF, Singh H, Storb U. Pip, a novel IRF family member, is a lymphoid-specific, PU.1-dependent transcriptional activator. Genes Dev. 1995;9(11):1377–87. . 7797077 10.1101/gad.9.11.1377

[24] Muhlethaler-Mottet A, Di Berardino W, Otten LA, Mach B. Activation of the MHC class II transactivator CIITA by interferon-gamma requires cooperative interaction between Stat1 and USF-1. Immunity. 1998;8(2):157–66. . 9491997 10.1016/s1074-7613(00)80468-9

[25] Pai RK, Askew D, Boom WH, Harding CV. Regulation of class II MHC expression in APCs: roles of types I, III, and IV class II transactivator. J Immunol. 2002;169(3):1326–33. . 12133955 10.4049/jimmunol.169.3.1326

[26] Fukai T, Nishiyama C, Kanada S, Nakano N, Hara M, Tokura T, et al Involvement of PU.1 in the transcriptional regulation of TNF-alpha. Biochem Biophys Res Commun. 2009;388(1):102–6. 10.1016/j.bbrc.2009.07.126 . 19646961

[27] Ito T, Nishiyama C, Nakano N, Nishiyama M, Usui Y, Takeda K, et al Roles of PU.1 in monocyte- and mast cell-specific gene regulation: PU.1 transactivates CIITA pIV in cooperation with IFN-gamma. Int Immunol. 2009;21(7):803–16. 10.1093/intimm/dxp048 . 19502584

[28] Ito T, Nishiyama C, Nishiyama M, Matsuda H, Maeda K, Akizawa Y, et al Mast cells acquire monocyte-specific gene expression and monocyte-like morphology by overproduction of PU.1. J Immunol. 2005;174(1):376–83. . 15611261 10.4049/jimmunol.174.1.376

