# Supplementary material for: Role of PU.1 in MHC Class II Expression via CIITA Transcription in Plasmacytoid Dendritic Cells
Source: PLoS One. 2016 Apr 22;11(4):e0154094. doi: 10.1371/journal.pone.0154094 (PMC4841550; doi:10.1371/journal.pone.0154094)
Supplement: S1 Table — (DOCX) [file pone.0154094.s001.docx]

Supplemental S1 Table. Nucleotide sequences of synthesized oligonucleotides for generation of reporter plasmids carrying the human CIITA-pIII promoters by using PCR and site-directed mutagenesis.

Primers for generation of various lengths of reporter plasmids (Fig. 3A and B)

Forward primer

-204_Kpn1-F 5’-atatggtaccCAATGATCACATGGTTTAGCCC-3’

-148_Kpn1-F 5’-ggccggtaccGCTTAAGGGAGTGTGGTAA-3’

-86_Kpn1-F 5’-aaggggtaccACCTTGCAGGGAGAGTTTT-3’

Reverse primer

+84_XhoI-R 5’-tatactcgagCTCCTCGTGCCCTCAGCTT-3’

* The inserted nucleotides are shown with small letters and *Kpn*I (GGTACC)- and *Xho*I (CTCGAG)-recognition sequences are underlined.

Primers for generation of mutant reporter plasmids (Fig. 3C)

Mut∆A

Forward 5’- CAGTCCACAGTAAGGggGTGgtAccAATTTCAGAGGTGTGGGG -3

Reverse 5’- CCCCACACCTCTGAAATTggTacCACccCCTTACTGTGGACTG -3

Mut∆B∆C

Forward 5’- AATTAGAGGGTGTTCAGgtACcGAAATCTGACCGCTTGGG -3

Reverse 5’- CCCAAGCGGTCAGATTTCgGTacCTGAACACCCTCTAATT -3

* The mutated nucleotides are shown with small letters.
