# Supplementary material for: Role of PU.1 in MHC Class II Expression via CIITA Transcription in Plasmacytoid Dendritic Cells
Source: PLoS One. 2016 Apr 22;11(4):e0154094. doi: 10.1371/journal.pone.0154094 (PMC4841550; doi:10.1371/journal.pone.0154094)
Supplement: S2 Table — (DOCX) [file pone.0154094.s002.docx]

Supplemental S2 Table. Nucleotide sequences of synthesized oligonucleotides for ChIP assay.

For human CIITApIII promoter,

forward primer (-230/-207) 5’- CAGACTTTCTGTGCAACTTTCTGT-3’

reverse primer (-124/-101) 5’- GATTTCTGTTTCTGAACACCCTCT-3’

For mouse CIITApIII promoter,

forward primer (-200/-180) 5’- CAGCTTCTGTGGTCACCCAAT-3’

reverse primer (-134/-125) 5’- TCCCTTAGCCACCACAGCTT-3’
